# When silence becomes violence: a perspective on institutional discourse, digital resistance, and educational justice in Temanggung, Indonesia

**DOI:** 10.3389/fsoc.2026.1814224

**Published:** 2026-05-29

**Authors:** Irwanto Irwanto

**Affiliations:** Film Department, School of Design, Bina Nusantara University, Jakarta, Indonesia

**Keywords:** collectivist communication, digital resistance, educational justice, Indonesia, institutional violence, trauma-informed pedagogy

## Abstract

Institutional language can function as a mechanism of educational violence, operating not through physical punishment but through systematic linguistic practices that silence vulnerable students. Drawing on analysis of the Temanggung school-burning case in Indonesia, where a 13-year-old bullying victim set fire to his school after months of institutional neglect, this perspective identifies three mechanisms of institutional silencing: dismissive minimization through psychiatric discourse, trauma invalidation through temporal displacement, and the criminalization of resistance through security discourse. Against the backdrop of 13,210 social media activities generated by this case, the evidence suggests that digital communities in collectivist societies develop counter-hegemonic resistance patterns that are fundamentally different from those observed in individualistic contexts, challenging dominant Western assumptions about digital polarization. This perspective introduces the Collective Resonance Communication Model (CRCM) as a new theoretical tool for understanding how cultural values shape digital discourse. The analysis concludes with a call for discourse justice as a precondition for educational justice, and practical implications for platform governance, educator training, and policy reform.

## 1 Background: language as the hidden weapon

On January 15, 2024, a 13-year-old student in Temanggung, Central Java, set fire to his school building. He had been bullied for months, reported it, and was told, in the language of authority, that he was lying.

This paper takes a clear position: what happened to that student before the fire was worse than the fire itself. The physical destruction of a building is visible, measurable, and legally actionable. The systematic destruction of a child’s credibility through institutional language, calling his distress “caper” (attention-seeking), dismissing his suffering as a family problem, and later characterizing his resistance as terrorism is invisible, deniable, and far more pervasive across Southeast Asian educational institutions.

We argue that institutional language is not merely a medium for communicating decisions: it is the mechanism through which educational violence is enacted. This distinction matters because it shifts responsibility away from isolated individuals (one negligent teacher, one callous principal) and toward the discursive structures that make such responses not only possible but institutionally protected. It also opens a different set of solutions, not only procedural reform but also what we term discourse justice: the fundamental transformation of how educational institutions speak to and about vulnerable students.

The institutional silencing documented in this case does not occur in a communication vacuum. When formal channels of accountability fail, communities increasingly turn to digital platforms as alternative arenas for articulating harm, demanding recognition, and mobilizing collective pressure. This shift reflects a broader transformation in how justice is negotiated in networked societies: digital media has become what Castells (2015) terms a space of autonomy, a communicative territory not fully controlled by institutional actors, in which counter-narratives can circulate without requiring institutional validation. For marginalized or silenced subjects, this autonomy is not merely convenient but structurally necessary: it provides the only platform on which their accounts of suffering can achieve visibility before they are filtered, dismissed, or reframed by the institutions responsible for the harm (Bennett and Segerberg, 2012; Gerbaudo, 2012; Rentschler, 2014; Banet-Weiser, 2018). In Indonesia, where vertical institutional hierarchies retain significant cultural authority, digital platforms have emerged as particularly important sites for horizontal solidarity and collective sense-making around cases of institutional failure (Lim, 2017; Anom et al., 2022). The Temanggung case crystallizes this dynamic: when institutional language silenced a child, 13,210 voices answered through social media. Understanding why requires not only discourse analysis but a theoretical account of how digital communities in collectivist societies organize around shared values of justice and child protection.

This perspective contributes three theoretical developments to the scholarship on educational justice in postcolonial Southeast Asian contexts (Francis et al., 2017): a framework for institutional silencing as educational violence, a reconceptualization of digital resistance in collectivist societies, and the Collective Resonance Communication Model (CRCM) as an alternative to Western echo-chamber theory. Together, these frameworks point toward practical interventions for educators, platform designers, and policymakers committed to genuinely inclusive educational environments.

A growing body of scholarship has examined institutional responses to school violence in Southeast Asian contexts (Handono et al., 2019; Kowalski et al., 2014; Nixon, 2014), as well as the dynamics of digital activism in Indonesian social media (Lim, 2017; Anom et al., 2022; Irwanto et al., 2025; Postill, 2018). However, three gaps remain underaddressed. First, existing studies of school bullying in Indonesia tend to focus on peer-to-peer dynamics and individual psychological outcomes, rather than on the institutional discursive mechanisms through which student accounts of harm are systematically delegitimized. Second, research on Indonesian digital activism has emphasized political and religious dimensions of online polarization but has not examined how collectivist cultural values structure digital discourse specifically around cases of educational injustice. Third, the available theoretical frameworks for digital discourse, predominantly derived from Western individualistic contexts, are poorly equipped to explain cross-cluster deliberation patterns that contradict echo-chamber predictions.

This perspective addresses these gaps by: (a) developing a framework for institutional silencing as a form of educational violence, grounded in Critical Discourse Analysis of the Temanggung case; (b) analyzing the structure rather than merely the volume of digital community response; and (c) introducing the Collective Resonance Communication Model (CRCM) as a culturally situated theoretical tool for understanding how collectivist values shape digital resistance. The novelty of this contribution lies not in documenting that institutional failure occurred, but in explaining precisely how institutional language enacts that failure, and how digital communities in collectivist societies develop coherent counter-hegemonic responses (Gramsci, 1971) that dominant Western theories predict should not exist.

## 2 The argument against individualistic readings

The dominant institutional response to cases like Temanggung follows a predictable logic: locate blame in an individual student’s pathology, a family’s dysfunction, or a single teacher’s failure. This individualistic reading is not accidental; it is a discursive strategy that protects institutional structures from accountability while appearing to take the incident seriously.

We reject this reading. Educational violence in Indonesia and across much of Southeast Asia is structural rather than individual. It is reproduced through hierarchical discourse patterns rooted in colonial-era administrative logics that position institutional authority as inherently credible and student voices as inherently suspect (Freire, 1970; Bourdieu, 1991; Spivak, 1988; Bhabha, 1994).

These patterns persist not because individual educators are malicious but because the discursive tools available to institutions, such as psychiatric diagnosis, temporal deferral, and security discourse, systematically favor institutional protection over student welfare. Institutional diagnosis of student distress as behavioral deviance rather than as a response to structural failure has been documented across diverse educational systems (Rogers, 2004; Mertens, 2007), while the use of deferral language to neutralize accountability is a recognizable feature of bureaucratic self-preservation in hierarchical organizations (Wodak and Meyer, 2009). In Indonesian educational contexts specifically, post-colonial administrative hierarchies continue to position institutional authority as inherently credible and student testimony as inherently suspect, a dynamic that Freire (1970) identified as foundational to oppressive pedagogical regimes and that Spivak (1988) later theorized as the structural incapacity of institutions to hear subaltern voices on their own terms.

Research on cyberbullying in Indonesian contexts finds that 35.6% of students experience peer harassment, yet institutional responses remain overwhelmingly inadequate (Handono et al., 2019; Kowalski et al., 2014). We argue that this inadequacy is not a failure of awareness but a function of institutional discourse: when the language available to educator’s frames student distress as individual pathology rather than institutional failure, an adequate response becomes structurally impossible.

## 3 How institutional silence operates: three mechanisms

Our critical discourse analysis of institutional responses in the Temanggung case, using Fairclough (2003) three-dimensional framework, reveals three primary mechanisms through which educational institutions silence vulnerable students. These mechanisms are not exceptional; they are the routine grammar of institutional self-protection.

### 3.1 Dismissive minimization through psychiatric discourse

Characterizing the student’s bullying reports as a “caper” (attention-seeking) is a clinical-sounding delegitimization that we term epistemic violence: the systematic denial of a student’s capacity to understand and articulate their own experience (Norton, 2013). This strategy operates through three sub-moves: credibility erosion (the student is an unreliable witness to their own suffering), responsibility displacement (the problem lies in insufficient parental affection, not institutional failure), and authority preservation (the institution retains credibility precisely by performing concern while avoiding accountability).

**Figure 1:**
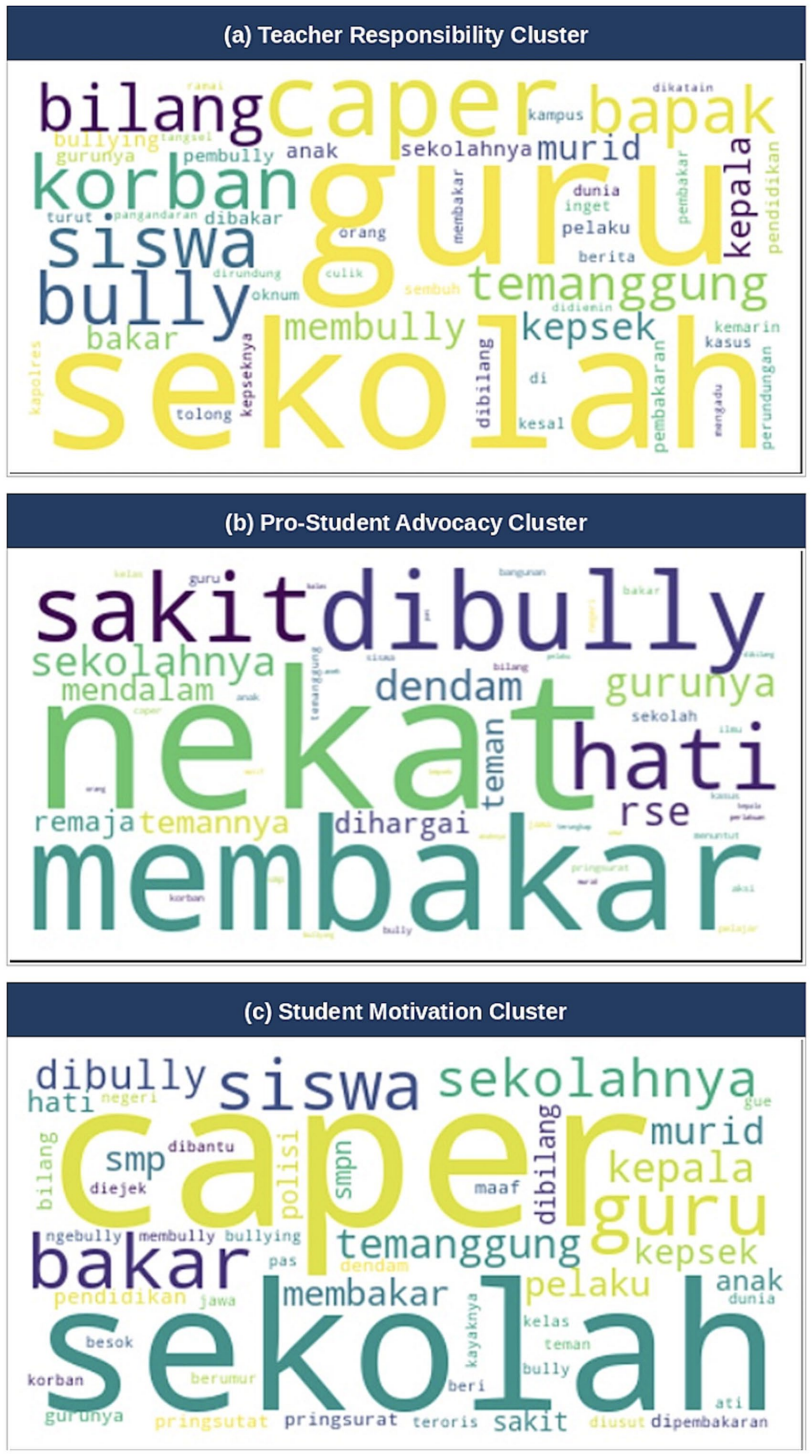
Wordcloud visualizations of dominant discourse terms across three primary clusters in the Temanggung case: **(a)** Teacher responsibility—institutional framing; **(b)** Pro-student advocacy—resistance and justice-oriented; and **(c)** Student motivation—emotional and contextual terms.

The word cloud in Figure 1, derived from discourse analysis of the Teacher Responsibility cluster (40.54% of total activity), shows how community respondents immediately identified and resisted this minimization—making terms such as “siswa” (student), “bully,” “korban” (victim), and “kepala” (principal/head) dominant and actively contesting the institution’s framing. The modal word “caper” itself became a viral signifier of institutional failure.

### 3.2 Trauma invalidation through temporal displacement

The institutional assertion that “anak-anak akan terbiasa dan dewasa dengan sendirinya” (children will get used to it and mature naturally) exemplifies a second mechanism: temporal displacement. By projecting resolution into an indefinite future governed by natural developmental processes, the institution acknowledges the existence of a problem while structurally avoiding any commitment to intervention. The future tense removes institutional agency; the claim to naturalness removes moral urgency.

Figure 1, from the Student Motivation cluster, shows the emotional vocabulary mobilized by the digital community to reconstruct student interiority, including “sakit” (pain), “dendam” (resentment), and “membakar” (burning). This vocabulary stands in sharp contrast to the temporal and administrative language of institutional responses, illustrating the discursive gap between student experience and institutional recognition.

### 3.3 Criminalization through security discourse

When dismissal and deferral fail to contain student resistance, institutional discourse escalates to criminalization. Describing a 13-year-old trauma victim as “seperti teroris” (like a terrorist) is not hyperbole: it is a calculated deployment of national security discourse that repositions the student as an external threat to be neutralized rather than an internal responsibility to be healed. This move is particularly effective in Indonesian contexts, where anti-terrorism frameworks carry significant cultural authority (Lim, 2017).

Figure 1 shows how the Pro-Student Advocacy cluster (17.03% of activity) directly engaged with this criminalization, with “teroris” emerging as a contested term that the community worked to reframe. The emergence of the hashtag #AnakBukanTeroris (Children Are Not Terrorists) as a policy advocacy tool demonstrates how community discourse can transform institutional language from a tool of suppression into a site of counter-argument.

## 4 Digital resistance in collectivist societies: a different model

The Temanggung case generated 13,210 social media activities from 5,893 accounts over 2 weeks. What is analytically significant is not the volume but the structure of this response—specifically, how it differs from patterns observed in digitally polarized, individualistic contexts.

**Figure 2:**
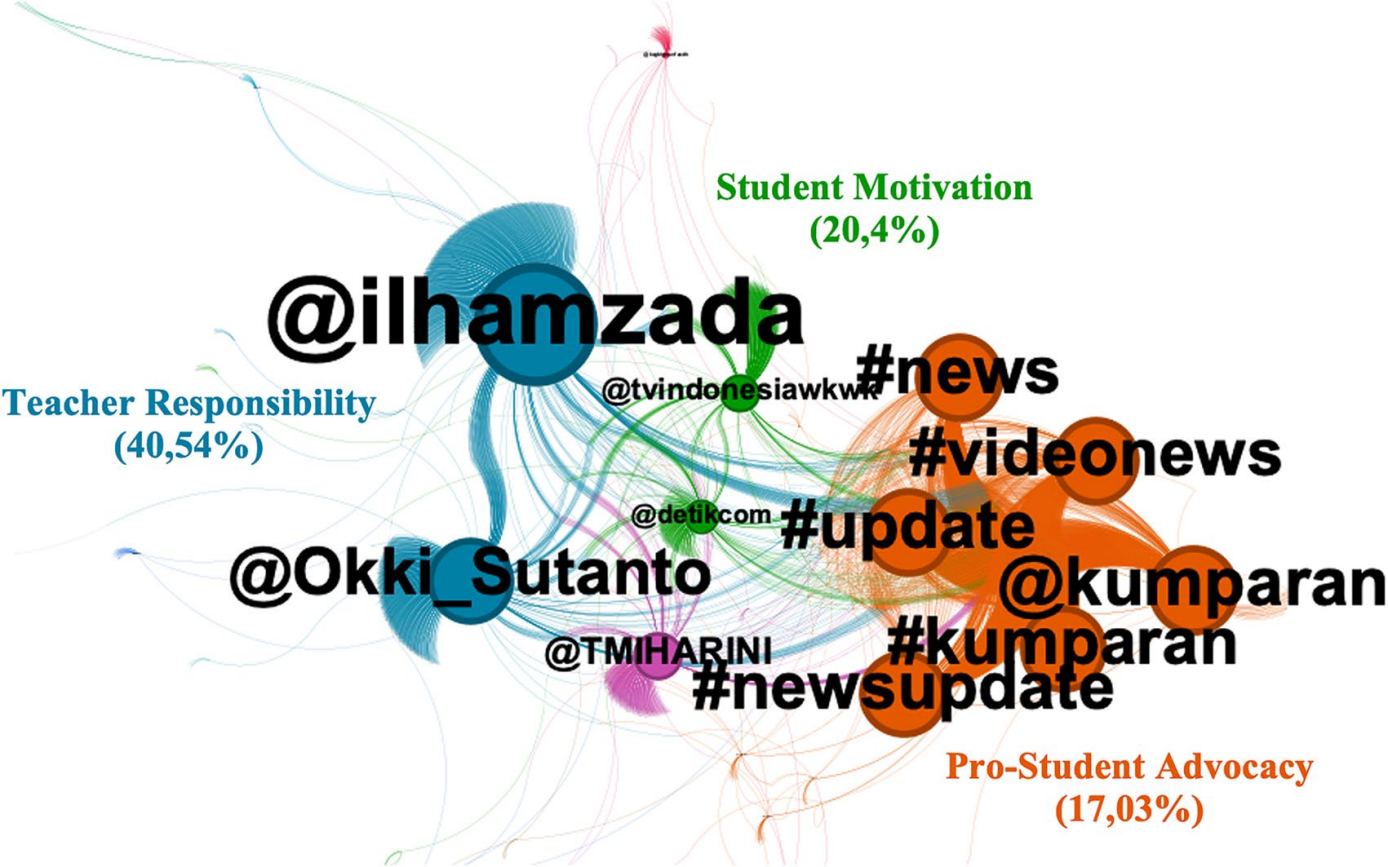
Overall network structure of Temanggung discourse showing three primary clusters—teacher responsibility (40.54%), student motivation (20.4%), and pro-student advocacy (17.03%)—and cross-cluster interactions. Node size represents engagement level; edge thickness indicates interaction strength.

As Figure 2 shows, three discourse clusters emerged, including Teacher Responsibility (40.54%), Student Motivation (20.4%), and Pro-Student Advocacy (17.03%), with cross-cluster interaction accounting for 25% of total activities. This rate is diagnostically important: in polarized digital environments, cross-cluster interaction typically remains below 5%, with users engaging primarily within ideologically homogeneous communities (Lim, 2017). The Temanggung case shows the opposite pattern.

This pattern is not coincidental: it reflects the operation of collectivist cultural values, specifically Indonesian traditions of musyawarah (collective deliberation) and gotong royong (cooperative problem-solving), in digital environments. Indonesian digital users, particularly in contexts of shared social concern, prioritize group coherence and collective sense-making over individual position-taking (Anom et al., 2022). The result is a form of digital discourse that challenges rather than reinforces polarization.

To understand why social media functioned as a space of justice in the Temanggung case, rather than merely a space of commentary, it is necessary to attend to the conditions that make institutional channels inadequate. When a child’s testimony is dismissed as attention-seeking, when parents are told the problem is theirs to solve, and when the student’s act of resistance is linguistically recast as terrorism, the formal channels of redress have not merely failed; they have been weaponized against the very person they were designed to protect. Under these conditions, digital platforms offer something that institutional channels structurally cannot: a communicative space in which the student’s account of suffering does not require institutional validation to achieve credibility.

The 13,210 social media activities generated in 2 weeks represent not simply public outrage but a collective epistemological act: the community asserted, against institutional denial, that the child’s suffering was real, that the institutional response was inadequate, and that responsibility lay with the system rather than the individual. The cross-cluster interaction rate of 25%, far exceeding the typically observed rate of below 5% in polarized digital environments (Lim, 2017), suggests that this collective work was genuinely deliberative: participants across discourse clusters engaged with perspectives not their own. What digital media provided, in this case, was not the content of justice but the conditions for its collective articulation, a platform on which 13,210 individual acts of recognition accumulated into something institutionally legible as demand. The temporal evolution of discourse from reactive emotion to structured policy advocacy, captured in the CRCM phases outlined in Section 5, further suggests that these conditions, when shaped by collectivist cultural values, can transform shared grief into governance critique.

This finding has significant theoretical implications. Dominant Western models of digital discourse predict that algorithmic filtering and user self-selection will produce echo chambers regardless of issue type (Sunstein, 2018). The Temanggung data suggest this prediction fails in collectivist cultural contexts. This analysis does not claim that Indonesian digital discourse is uniformly deliberative. Lim (2017) has documented significant tribal nationalism in Indonesian social media, but it is argued that educational justice cases, which invoke shared values around child protection and collective responsibility, activate different patterns of engagement.

## 5 Explaining cross-cluster dialogue

To account for these patterns, this perspective introduces the Collective Resonance Communication Model (Irwanto et al., 2025): a framework for understanding how information spreads and generates meaning within collectivist digital communities engaged in institutional critique.

The CRCM identifies five interconnected components. Central Resonators are key actors, including mainstream media and influential figures, who achieve content adoption rates of 30%–35% by aligning their framing with shared community values rather than simply broadcasting to passive audiences. Resonance Nodes are bridge actors (approximately 10%–15% of participants) who facilitate cross-cluster information flow, achieving average engagement rates of 2.24–3.18 activities per account. Resonance Waves are temporal patterns characterized by peak engagement within 4–6 h of key discursive events, followed by sustained deliberation for 24–48 h. Distribution Patterns are the formalized pathways through which traditional hierarchies, such as media organizations, public intellectuals, and community leaders, adapt to digital environments while maintaining cultural legitimacy. Finally, Cross-Cluster Interaction rates of 15–25% represent the model’s most diagnostically distinctive feature: the maintenance of inter-perspectival dialogue that prevents discourse calcification.

The CRCM also captures the temporal evolution of digital discourse in the Temanggung case. Analysis reveals five phases: initial shock and exploratory connection (0–6 h); narrative construction and hashtag emergence, notably #SuaraKorban (Victim’s Voice), in 6–24 h; institutional critique and the rise of #SekolahGagal (Failed School) in 24–48 h; systemic policy analysis at peak cross-cluster interaction in 48–96 h; and solution generation centered on #AnakBukanTeroris in 96 + hours. Network density evolved from 0.32 (loose, exploratory) to 0.48 (structured, solution-focused) across this period, evidence that digital deliberation in this context became more rather than less coherent over time.

This trajectory challenges the common assumption that social media engagement necessarily degenerates from nuanced discussion into inflammatory reaction. In the Temanggung case, the reverse occurred: what began as reactive emotion transformed into sophisticated policy advocacy. The evidence suggests that the CRCM explains this outcome by revealing how collectivist values structure digital discourse toward consensus-building rather than conflict escalation.

## 6 Implications: toward discourse justice

### 6.1 For educational institutions

The mechanisms of institutional silencing identified here are not unusual: they are the default grammar of institutional self-protection in hierarchical educational systems. Changing them requires not procedural adjustment but what is termed discourse justice: a fundamental reorientation of institutional communication that treats student testimony as presumptively credible, resists pathologizing language, and commits to immediate rather than temporally deferred accountability.

Practically, this means mandating trauma-informed communication protocols, developing educator training that explicitly addresses the linguistic strategies through which institutional violence operates, and creating formal channels through which student testimony reaches decision-makers without being filtered by the same institutional apparatus that produced the original harm (Selwyn, 2019).

### 6.2 For platform governance

The CRCM reveals both the potential and the vulnerability of digital discourse in collectivist contexts. Cross-cluster interaction rates that support democratic deliberation can be suppressed by algorithmic systems optimized for engagement maximization, which tend to amplify emotionally reactive content over deliberative exchange. Platform governance in Southeast Asian contexts should support rather than undermine collectivist deliberation patterns. This may require culturally specific algorithmic calibration rather than universal global standards (Van Dijck, 2024).

At the same time, the Temanggung case illustrates the risk of trauma commodification when individual suffering becomes viral content (Banet-Weiser, 2018). Platform governance frameworks must develop stronger protections for minors whose experiences enter public digital discourse, including mechanisms for content removal upon request from affected parties, regardless of engagement metrics.

### 6.3 For researchers

This perspective calls for comparative research across Southeast Asian educational contexts (Welch, 2011) to test whether the CRCM patterns identified here generalize beyond Indonesia and for longitudinal research to track whether digital resistance of the Temanggung type produces lasting institutional change. It also calls for intersectional analysis examining how gender, ethnicity, and socioeconomic position modulate experiences of institutional silencing (Bhopal and Henderson, 2021): the Temanggung case involved a male student in a semi-rural context, and these findings cannot be assumed to generalize uniformly across all student identities.

## 7 Conclusion: the grammar of justice

The Temanggung school-burning case teaches us something that individual psychological interventions cannot address: that educational institutions can be structurally violent in their most ordinary operations, through the routine language of administration and professional authority. When a child is told that their suffering is attention-seeking, that time will resolve what institutions will not, and that their resistance marks them as a terrorist, the institution has not merely failed; it has acted. Language is the action.

As argued throughout this perspective, understanding this violence requires new theoretical tools: institutional silencing as a framework for the mechanisms of discursive harm; the CRCM as a framework for understanding the distinctive counter-hegemonic potential of digital communities in collectivist societies; and discourse justice as the goal toward which educational reform must orient itself.

The 13,210 voices that responded to one child’s act of desperation in Temanggung demonstrate that communities possess both the knowledge and the will to demand better educational institutions. What they require is not rescue by external experts but institutional structures that genuinely hear them, and researchers, educators, and policymakers with the theoretical vocabulary and political courage to insist on that hearing. Educational justice begins with the grammar of how institutions speak.

## Data Availability

The original contributions presented in the study are included in the article/supplementary material, further inquiries can be directed to the corresponding author.
